# The association between lactate dehydrogenase to serum albumin ratio and in-hospital mortality in patients with pulmonary embolism: a retrospective analysis of the MIMIC-IV database

**DOI:** 10.3389/fcvm.2024.1398614

**Published:** 2024-06-19

**Authors:** Jingjing Hu, Yidan Zhou

**Affiliations:** Department of Emergency Medicine, Hangzhou Third People’s Hospital, Hangzhou, China

**Keywords:** lactate dehydrogenase, albumin, pulmonary embolism, venous thrombosis, acute pulmonary embolism

## Abstract

**Background:**

Lactate dehydrogenase (LDH) and albumin (ALB) were found to be significantly correlated with mortality in pulmonary embolism (PE) patients. However, data regarding the LDH/ALB ratio (LAR) in patients with acute PE are scanty. Therefore, the aim of this study was to investigate the association between LAR and the risk of mortality in patients with acute PE.

**Methods:**

A retrospective cohort study was conducted on patients with acute PE represented in the Medical Information Mart for Intensive Care IV (MIMIC-IV). A receiver operating characteristic (ROC) curve analysis and calibration curve were used to assess the accuracy of the LAR for predicting mortality in patients with acute PE. We utilized Cox regression analysis to determine adjusted hazard ratios (HR) and 95% confidence interval (CI). Survival curves were used to evaluate a connection between the LAR and prognosis in patients with acute PE.

**Results:**

The study comprised 581 patients, and the 30-day all-cause mortality rate was 7.7%. We observed a higher LAR in the non-survival group compared to the surviving group (21.24 ± 21.22 vs. 8.99 ± 7.86, *p* < 0.0001). The Kaplan–Meier analysis showed that patients with an elevated LAR had a significantly lower likelihood of surviving the 30-day mortality compared to those with a low LAR. Cox regression analysis showed that LAR (HR = 1.04, 95% CI: 1.03–1.05) might have associations with 30-day mortality in patients with acute PE. This result was supported by sensitivity analyses. According to the results of the ROC curve analysis, the LAR's prediction of 30-day mortality in patients with acute PE yielded an area under the ROC curve of 0.73. A calibration curve showed LAR is well calibrated.

**Conclusion:**

Our research suggests LAR monitoring may be promising as a prognostic marker among patients with acute PE.

## Introduction

Pulmonary embolism (PE) is a serious disease, exhibiting high morbidity and mortality rates, and stands as the third most fatal cardiovascular disease, surpassed only by acute myocardial infarction (MI) and stroke (1–3). It impacts millions of individuals worldwide, representing a substantial contributor to morbidity, mortality, and escalating healthcare expenditure globally. Despite considerable progress in the diagnosis and treatment of acute pulmonary embolism (acute PE) in recent years, the incidence of this condition continues to rise year on year (3). In addition, despite a global trend toward decreasing fatality rates of PE, the associated mortality proportions continue to appear high (4). In the acute phase, a wide clinical spectrum is observed, ranging from asymptomatic PE to cardiovascular collapse and mortality. Therefore, it is essential to conduct accurate assessments and risk stratification of patients with acute PE to assist clinicians in making optimal treatment decisions. According to relevant studies, the Pulmonary Embolism Severity Index (PESI) score is the most commonly used method for predicting short-term mortality in patients with acute PE. However, the PESI score involves numerous variables with varying weights and is complex to calculate, making it less widely accepted among emergency medical personnel (5). Hence, the simplified Pulmonary Embolism Severity Index (sPESI) has been developed to predict the risk of death in patients with acute PE (6). Furthermore, the strengths of both the PESI and sPESI primarily lie in identifying patients with low- to moderate-risk acute PE, but their ability to identify patients with moderate- to high-risk acute PE is limited (5). Therefore, there is an urgent need for simple and reliable methods to assist clinicians in the early assessment of patients with acute PE in the intensive care unit (ICU) and to enhance the accuracy of existing prognosis predictions for critically ill patients.

Venous thrombosis (VTE) primarily involves three elements: stagnant blood flow, vascular injury, and hypercoagulability of the blood (7). Studies have shown that immune response and inflammation play an indispensable role in the above pathophysiological processes (8). Vascular endothelial cells (VECs) play essential roles in the pathogenesis of VTE. Previous studies have documented that VTE induces VEC injury, the accumulation of inflammatory substances, blood hypercoagulation, and worsened thromboembolism, resulting in severe health (9–11). Lactate dehydrogenase (LDH) is regarded as a biomarker of disease activity and tissue damage. In some previous studies, an elevated LDH level was identified to be independently associated with VTE (12–15). Moreover, in recent years, a wealth of research has suggested that inflammation plays a crucial role in the pathophysiological processes of VTE (16). Although the relationship between inflammation and clinical venous thrombosis is not clearly known, it is speculated that inflammation of the vessel wall initiates thrombus formation in an intact vein and that inflammation and coagulation systems are coupled by a common activation pathway (17). Serum albumin (ALB) is an acute-phase protein primarily synthesized in the liver and serves as a reliable indicator of nutritional status (18). ALB performs several crucial physiological functions, including exerting antithrombotic effects, inhibiting platelet aggregation, and promoting anticoagulation (19). Furthermore, ALB can influence the thrombotic triad and regulate abnormal coagulation through various mechanisms (20). Research indicates that low ALB levels serve as an indicator of poor prognosis in hospitalized patients (21, 22). Furthermore, certain studies have identified ALB as an independent predictor of mortality associated with PE (23–27). One hypothesis is that elevated LDH and reduced albumin might be indicative of a hyperinflammatory or hypercoagulable state (20, 23, 28, 29). Recently, the LAR (LDH/ALB ratio) has been found to be significantly correlated with the mortality rates of sepsis and sepsis-associated acute kidney injury (30, 31). Therefore, it is essential to investigate the correlation between LAR and acute PE.

However, data regarding LAR in acute PE patients are scanty. We hypothesized that an increased LAR could be associated with a poor prognosis in patients with acute PE. Accordingly, we sought to explore the relationship between LAR and acute PE in critically ill patients.

## Materials and methods

### Data source

Based on primary data obtained from the comprehensive Medical Information Mart for Intensive Care IV (MIMIC-IV) database (version 2.2), this retrospective observational study was conducted (32). The MIMIC-IV database includes data for each medical record pertaining to patients admitted to the ICU or emergency room at the Beth Israel Deaconess Medical Center between 2008 and 2019. Acknowledged as one of the most voluminous and frequently engaged databases in intensive care medicine, it offers crucial resources for research and analytic purposes. The establishment of the database was approved by the institutional review boards of the Massachusetts Institute of Technology (Cambridge, MA, USA) and Beth Israel Deaconess Medical Center (Boston, MA, USA). To maintain ethical standards and protect patient privacy, the data utilized in this study underwent de-identification, with all necessary precautions implemented to safeguard patient confidentiality. The first author (Jingjing Hu, certification ID: 52583254) was authorized to use the MIMIC-IV database after completing the National Institutes of Health's online education program. The requirement for informed consent was waived by the ethical committee at Beth Israel Deaconess Medical Center, given the de-identified nature of the data.

### Study population

All eligible patients diagnosed with acute PE based on ICD codes (ICD-9: 41511, 41512, 41519, 67380–67384; ICD-10 code: I26, I260, I2601, I2609, I269, I2690, I2693, I2699) from the MIMIC-IV database were included as participants in this study. The inclusion criteria comprised adult patients (aged ≥18 years) diagnosed with acute PE. For individuals with repeated admissions, only data from their first admission were considered. The exclusion criteria included ICU readmission, individuals lacking data on ALB or LDH within 24 h, missing data for calculating important metrics such as PESI and sPESI, and those with insufficient follow-up information. As a result, this study comprised only 581 patients.

### Data extraction

Structured Query Language data extraction tool (pgAdmin 4) was used to extract data in the first 24 h of admission: (1) demographic variables: age, sex, race, insurance status, and initial admission unit; (2) vital signs: temperature, respiratory rate (RR), heart rate, systolic blood pressure (SBP), diastolic blood pressure (DBP), mean arterial pressure (MAP), and oxygen saturation levels (SpO_2_) were recorded on the first day of admission; (3) comorbidities: MI, heart failure (HF), chronic pulmonary disease, diabetes mellitus (DM), renal disease, and liver disease; (4) laboratory tests were performed within the initial 24 h after admission, including hematocrit level, white blood cell (WBC) count, platelet count, serum potassium level, serum sodium level, hemoglobin level, blood glucose level, serum bicarbonate level, serum calcium level, serum chloride level, serum urea nitrogen level (BUN), serum creatinine level (SCR), anion gap, prothrombin time (PT), partial thromboplastin time (PTT), international normalized ratio (INR), LDH, ALB, and other laboratory markers. If a variable was measured multiple times within the previous 24 h, the mean value was used; (5) Simplified Acute Physiology Score II (SAPS II) score, PESI score, sPESI score, and Sequential Organ Failure Assessment (SOFA) score were used to assess the severity of illness upon admission; (6) the use of vasoactive drugs, continuous renal replacement therapy (CRRT) and mechanical ventilation (MV) during hospitalization; (7) the duration of ICU stays, overall length of hospital stay, and cases of 30-day death during hospitalization. LAR was calculated from the LDH (U/L)/ALB (g/L) ratio.

### Endpoints

The endpoint was 30-day all-cause mortality.

### Statistical analysis

The basic clinical characteristics of patients were analyzed according to the death and survival groups. Normal distribution was assessed using the Shapiro–Wilk test. Categorical variables were presented as numbers and percentages (%) and were compared using the chi-square test. Continuous variables were presented as mean ± standard deviation (SD) for variables with normal distribution or as median [interquartile range (IQR)] for variables without normal distribution and compared using either the Student *t*-test or Mann–Whitney *U* test, respectively. Survival curves were generated using the Kaplan–Meier method and compared using the log-rank test.

Cox proportional hazard models were constructed to test the associations between 30-day mortality, with results expressed as hazard ratios (HR) with 95% confidence intervals (CIs). We used multivariate Cox regression models to estimate the relationship between LAR and 30-day mortality in patients with acute PE. Model I adjusted for nothing. Model II adjusted for age, sex, race, and insurance. Model III adjusted for use of vasoactive drugs, use of CRRT, use of MV, and ICU management at admission. Model IV adjusted for SAPS II score and PESI score. A time-dependent receiver operating characteristic (ROC) curve analysis was used to assess the predictive ability of LAR, PESI score, and sPESI score for 30-day mortality. Through this analysis, the sensitivity and specificity of each index and the area under the ROC curve (AUC) were computed. The optimal cut-off value of LAR was ascertained by the Youden index. The calibration curve was used to evaluate the agreement between the number of observed and expected deaths predicted by LAR. A calibration curve close to the ideal *y* = *x* line indicates good calibration.

Finally, because non-ICU patients lacked crucial data, our final analysis only included patients admitted to the ICU. Therefore, we conducted a sensitivity analysis. This analysis included 338 non-ICU patients with acute PE with LDH and ALB data available within 24 h. Sensitivity analyses were also conducted on 338 patients with acute PE who were not admitted to the ICU. Furthermore, because active tumors have a significant impact on patient survival, we excluded this subset of patients and conducted sensitivity analyses (*n* = 393). We used Cox regression models to estimate the relationship between LAR and 30-day mortality in patients with acute PE. Data analyses were conducted using Stata 16.0. Statistical significance was defined as a two-tailed *p*-value <0.05.

## Results

### Study population and basic clinical characteristics

**Figure 1:**
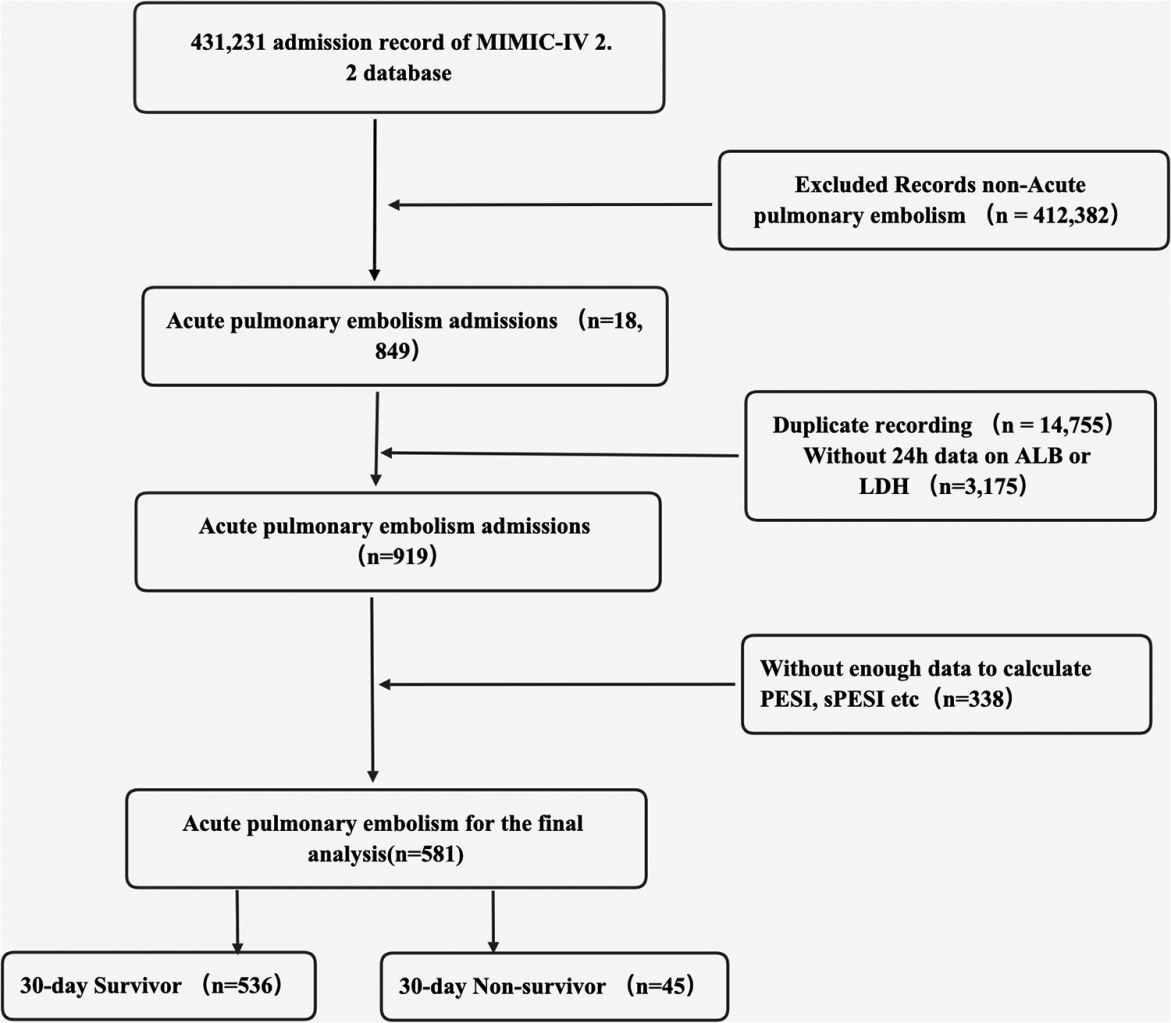
The flow chart of patient selection.

Overall, a total of 581 participants were included in the final study according to the inclusion and exclusion criteria (Figure 1). The basic clinical characteristics of all participants were shown in Table 1. The 30-day mortality for patients with acute PE in this study was 7.7%. Patients in the non-survival group had higher LAR (21.24 ± 21.22 vs. 8.99 ± 7.86, *p *< 0.0001) and age (71 vs. 62 years, *p* = 0.001). The patients in the non-survival group were more likely to have a history of MI (22.2% vs. 9.3%, *p* = 0.006), HF (28.9% vs. 6.4%, *p* = 0.034), DM (28.9% vs. 15.9%, *p* = 0.025), liver disease (33.3% vs. 13.1%, *p* < 0.0001), and cancer (37.8% vs. 31.9%, *p* = 0.041). However, no statistical differences were found in the history of chronic pulmonary disease, renal disease, and sepsis (all *p* > 0.05). Patients in the non-survival group had a higher APSII score, PESI score, sPESI score, and SOFA score (all *p* < 0.05). Patients in the non-survival group were more likely to use vasopressin (37.9% vs. 6.9%, *p* < 0.0001) and MV (55.6% vs. 28.5%, *p* < 0.0001), while no difference was observed in CRRT (4.4% vs. 1.7%, *p* = 0.191).

**Table 1:** Baseline characteristics of participants.

Variables	Total (*n* = 581)	30-day survivors (*N* = 536)	30-day non-survivors (*N* = 45)	*p*-value
LDH/ALB	9.94 ± 10.10	8.99 ± 7.86	21.24 ± 21.22	<0.0001
Male, *n* (%)	297 (51.1%)	278 (51.9%)	19 (42.2%)	0.214
Age, years	64 (52–74)	62 (52–73)	71 (66–80)	0.001
Insurance, *n* (%)	279 (48.0%)	252 (47.0%)	27 (60%)	0.094
Ethnicity, *n* (%)				0.120
White	384 (66.1%)	359 (67.0%)	25 (55.6%)	
Other	197 (33.9%)	177 (33.0%)	20 (44.4%)	
ICU management at admission	538 (92.6%)	498 (92.9%)	40 (88.9%)	0.322
Vital signs				
HR (beats/min)	92 (80–104)	91 (80–104)	93 (85–104)	0.3075
SBP (mmHg)	115 (105–127)	116 (105–127)	108 (103–117)	0.0210
DBP (mmHg)	65 (58–71)	65 (59–72)	58 (54–64)	0.0001
Mean arterial pressure (mmHg)	78 (71–85)	78 (72.85)	71 (67–78)	0.0001
RR (times/min)	20 (17–23)	20 (17–23)	22 (19–26)	0.0001
Temperature (°C)	36.8 (36.6–37.1)	36.8 (36.5–37.1)	36.8 (36.6–17.1)	0.8284
SpO_2_ (%)	97 (95–98)	97 (96–98)	96 (93–98)	0.0201
Comorbidities, *n* (%)				
Myocardial infarction	60 (10.3%)	50 (9.3%)	10 (22.2%)	0.006
Congestive heart failure	101 (17.4%)	88 (16.4%)	13 (28.9%)	0.034
Chronic pulmonary disease	136 (23.4%)	126 (23.5%)	10 (22.2%)	0.845
Diabetes	98 (16.9%)	85 (15.9%)	13 (28.9%)	0.025
Liver disease	85 (14.6%)	70 (13.1%)	15 (33.3%)	<0.0001
Malignancy	188 (32.4%)	171 (31.9%)	17 (37.8%)	0.041
Renal disease	85 (14.6%)	74 (13.8%)	11 (24.4%)	0.052
Sepsis	114 (19.6%)	101 (18.8%)	12 (26.7%)	0.215
Laboratory results	31.1 (27.3–35.6)	31.2 (27.4–35.6)	29.4 (26.7–34.9)	0.2125
Hematocrit (%)	10.1 (8.8–11.8)	10.2 (8.9–11.9)	9.6 (8.3–11.1)	0.0261
Hemoglobin (g/dl)	205 (140–286.5)	207 (141–286)	187 (95–287)	0.1007
Platelets (10^3^/µl)	10.8 (7.45–14.7)	10.5 (7.3–14.2)	16.2 (10.2–22.2)	<0.0001
WBC (10^3^/μl)	14 (12–16)	14 (12–16)	16 (14–19.5)	<0.0001
Anion gap (mmol/L)	23 (20–26)	23.5 (20–26)	20 (18–23)	0.0002
Bicarbonate (mmol/L)	18 (12–28)	17 (12–26)	33 (26.5–52)	<0.0001
BUN (mg/dl)	8.2 (7.8–8.7)	8.2 (7.9–8.7)	8.1 (7.5–8.6)	0.2334
Calcium (mmol/L)	104 (101–108)	104 (101–108)	104 (98–110)	0.8258
Chloride (mmol/L)	0.95 (0.7–1.35)	0.9 (0.7–1.25)	1.35 (0.95–195)	<0.0001
Creatinine (mg/dl)	125 (105–158)	124 (104–153)	162 (111–202)	0.0002
Glucose (mmol/L)	138 (136–141)	138 (136–141)	139 (134–144)	0.4508
Sodium (mmol/L)	4.1 (3.8–4.6)	4.1 (3.8–4.5)	4.4 (3.9–5.0)	0.0383
Potassium (mmol/L)	1.3 (1.15–1.5)	1.3 (1.15–1.5)	1.45 (1.35–1.73)	0.0001
INR	14.5 (13.1–16.5)	14.4 (13–16.3)	16.1 (14.4–18.7)	0.0005
PT (s)	33.5 (28.8–55.8)	32.8 (28.6–53.1)	56.7 (37.2–88.9)	0.0001
PTT (s)	238 (187–335)	230 (186–321)	386 (254–673)	<0.0001
LDH (U/L)	37 (31–42)	37 (32–42)	29 (25–34)	<0.0001
ALB (g/L)				
Therapy, *n* (%)				
Vasopressin	54 (9.3%)	37 (6.9%)	17 (37.9%)	<0.0001
CRRT	11 (1.9%)	9 (1.7%)	2 (4.4%)	0.191
Ventilation	178 (30.6%)	153 (28.5%)	25 (55.6%)	<0.0001
Scores				
SOFA	4 (2–6)	3 (2–6)	8 (4–11)	<0.0001
APSII	35 (27–44)	34 (26–43)	52 (40–63)	<0.0001
PESI	96 (74–121)	94.5 (74–119)	123 (79–148)	0.001
I, *n* (%)	84 (14.5%)	80 (14.9%)	4 (8.9%)	
II, *n* (%)	135 (23.2%)	126 (23.5%)	9 (20.0%)	
III, *n* (%)	132 (22.7%)	128 (23.9%)	4 (8.9%)	
IV, *n* (%)	106 (18.2%)	99 (18.5%)	7 (15.6%)	
V, *n* (%)	123 (21.3%)	103 (19.2%)	21 (46.7%)	
sPESI	1 (0–2)	1 (0–2)	1 (1–2)	0.007
Outcomes, days				
Los-ICU	2.2 (1.2–4.8)	2.1 (1.2–4.6)	3.4 (1.8–8.2)	0.0186
Los-hospital	9.4 (5.7–17.1)	9.5 (5.8–17.2)	8.1 (3.5–15.2)	0.034

### Relationship between LAR and 30-day all-cause mortality

When analyzed as a continuous variable, LAR was associated with 30-day mortality. As exhibited in Table 2, univariate analysis revealed that LAR (HR = 1.04, 95% CI: 1.03–1.05) might have associations with 30-day mortality in patients with acute PE. After adjusting for confounders including age, sex, race, and insurance, the LAR level was associated with an increased risk of 30-day mortality in patients with acute PE (HR = 1.04, 95% CI: 1.03–1.06). Model III adjusted for use of vasoactive drugs, use of CRRT, use of MV, and ICU management at admission, and the LAR level was associated with an increased risk of 30-day mortality in patients with acute PE (HR = 1.04, 95% CI: 1.03–1.05). Model IV adjusted for SAPS II score and PESI score, and the LAR level was associated with an increased risk of 30-day mortality in patients with acute PE (HR = 1.04, 95% CI: 1.02–1.05).

**Table 2:** HRs for 30-day mortality based on LAR in patients with acute pulmonary embolism.

LAR	Crude model HR (95 CI)	Model I HR (95 CI)	Model III HR (95 CI)	Model II HR (95 CI)
30-day mortality	1.04 (1.03–1.05)	1.04 (1.03–1.06)	1.04 (1.03–1.05)	1.04 (1.02–1.05)
Sensitivity analysis				
Without ICU admission (*n* = 338)	1.12 (1.08–1.17)	1.20 (1.11–1.29)	1.20 (1.11–1.29)	—
Without active tumors (*n* = 393)	1.05 (1.03–1.06)	1.05 (1.03–1.06)	1.04 (1.03–1.06)	1.04 (1.02–1.05)
All acute PE (*n* = 919)	1.05 (1.04–1.06)	1.05 (1.04–1.06)	1.05 (1.03–1.06)	—

Crude model: adjusted for nothing. Model I: adjusted for age, sex, race, and insurance. Model II: adjusted for use of vasoactive drugs, use of continuous renal replacement therapy, use of mechanical ventilation, and ICU management at admission. Model III: adjusted for SAPS II score and PESI score.

### Analysis of ROC curves and calibration curve

**Figure 2:**
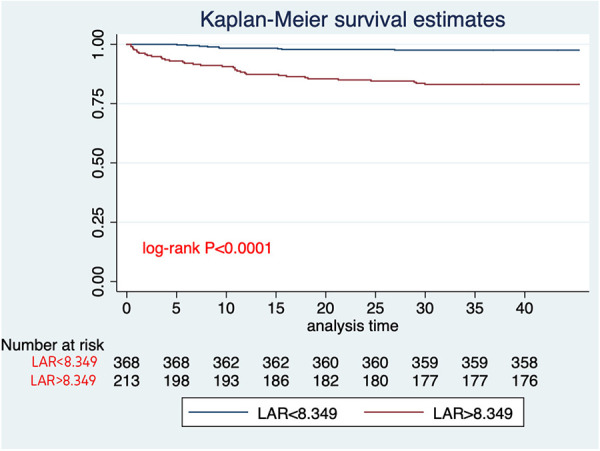
Kaplan–Meier curves of the LAR for 30-day mortality of patients with acute PE.

**Figure 3:**
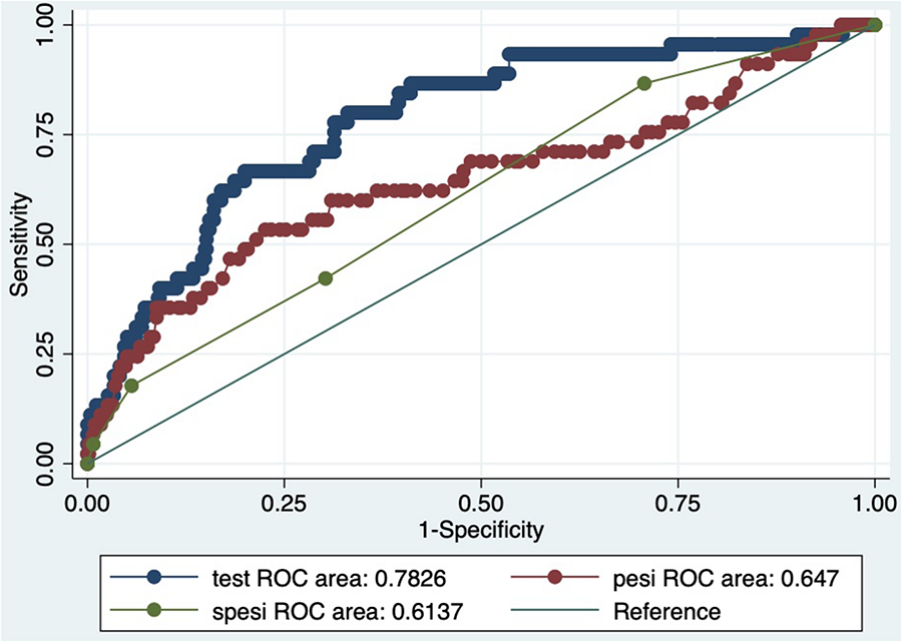
ROC curve.

**Figure 4:**
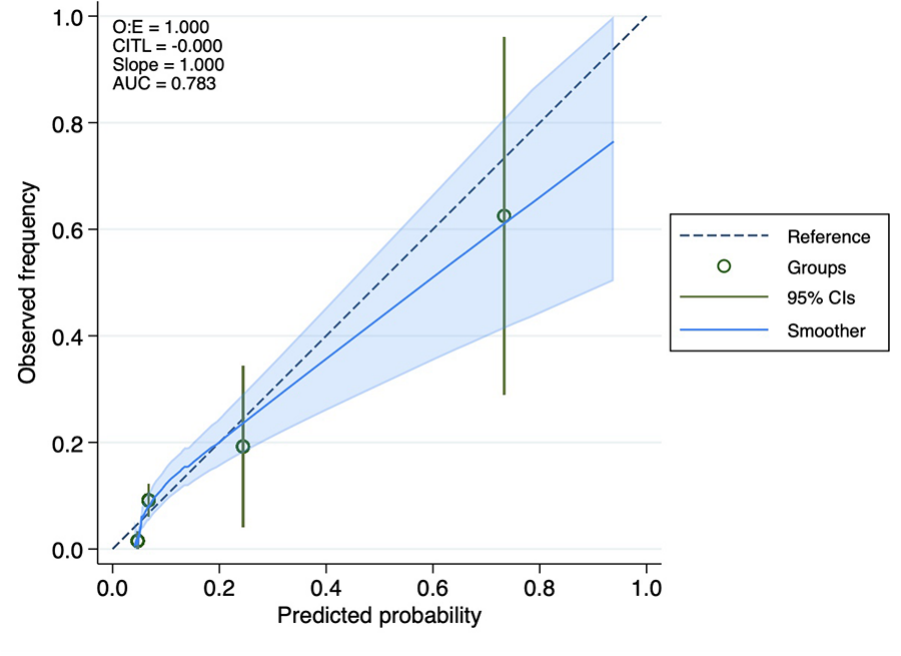
Calibration curve of the LAR.

The analysis of ROC curves demonstrated that LAR exhibited a significant predictive ability for 30-day mortality, with an optimal threshold of 8.349, a sensitivity of 80%, and a specificity of 67%. It is noteworthy that the predictive performance of LAR closely resembled that of PESI score, and sPESI score (PESI score AUC = 0.65; sPESI score AUC = 0.56). LAR is well calibrated; however, at higher score ranges, mortality may be underestimated (Figures 2–4).

### Sensitivity analysis

We conducted a sensitivity analysis, including 338 non-ICU patients. A total of 919 patients were included in the analysis, among which the overall mortality rate was 5.5% (51 deaths), with a mortality rate of 7.7% (45 deaths) among patients admitted to the ICU and 1.8% (6 deaths) among patients not admitted to the ICU. Furthermore, we conducted sensitivity analyses on patients with acute PE (*n* = 393) who were managed in the ICU without active tumors and on patients who were managed outside the ICU without active tumors (*n* = 338). The sensitivity analysis supported our conclusion that LAR is associated with 30-day mortality in patients with acute PE (Table 2).

## Discussion

In this study, we investigated the prognostic values of LAR for the 30-day mortality of patients with acute PE using data from the MIMIC-IV database. To the best of our knowledge, our study is the first to investigate the association between LAR and all-cause mortality in patients with acute PE. In this retrospective study, we analyzed 581 patients with acute PE from the MIMIC-IV database and found that the non-survival group had significantly higher serum LAR levels than the survival group. Serum LAR levels were independently positively associated with 30-day mortality in patients with acute PE. The AUC values of LAR for diagnosing the risk of 30-day mortality in acute PE were 0.73. The findings suggested that LAR monitoring may be promising as a prognostic marker among patients with acute PE. However, our conclusions still need to be verified in multicenter, prospective cohort studies.

Despite advancements in diagnostic and treatment, PE persists as a global public health concern, with annual incidence rates continuing to rise over time (3). While the global fatality rates of PE tend to decrease, the mortality proportions associated with PE still appear to be high, accounting for up to 0.2%–0.4% of all deaths (4). The international Registro Informatizado Enfermedad Tromboembólica (RIETE) registry study revealed that the all-cause mortality rate for patients with PE was 4.9%–6.6% at 30 days (33). In our study, the 30-day mortality of patients with acute PE was 7.7%. A 30-year nationwide population-based cohort study involving 128,223 patients found that the mortality risk for patients with acute PE within the first 30 days was 31% (34). On the other hand, a study conducted at Mayo Clinic reported a similar 30-day mortality of 7.7%, consistent with our findings (35). Mortality among patients with acute PE vary across studies. This variability may be attributed to differences in population characteristics, available medical resources, and the severity of PE among the various studies.

LDH is regarded as a biomarker of disease activity and tissue damage. Alkindi et al. found a higher LDH level in patients with VTE compared with the controls (*p* < 0.05) (15). Abdel-Razeq et al. conducted a retrospectively study and found that a high LDH level was an important factor that may increase the risk of VTE [Odds Ratio (OR) = 5.82] (12). Similarly, Chen et al. discover that a high LDH level was significantly associated with VTE (OR = 2.441). The pathophysiological mechanism of how LDH causes VTE has not been fully elucidated. VECs serve key roles in the pathogenesis of VTE. Previous studies have documented that VTE induces VEC injury, the accumulation of inflammatory substances, blood hypercoagulation, and worsened thromboembolism, resulting in severe health consequences (9–11). The impairment of VECs may compromise the integrity of blood vessels, potentially leading to bleeding, which can impact the anticoagulant therapy in VTE (36). Some studies showed lactate (LA) may serve an important role in VEC damage (37, 38). VECs uptake glucose from peripheral blood and, through the catalytic activity of various enzymes such as glycogen and pyruvate kinase, convert glucose to tricarboxylic acid. This process serves as an energy source for cells. Apart from this, pyruvate can also be converted to LA by lactic acid dehydrogenase A (LDHA) (39–42). However, LA does not directly provide energy to cells; it must first be converted by lactic acid dehydrogenase B (LDHB) to pyruvate (43). Some studies suggest that elevated LA levels contribute to VEC aging, potentially associated with an increased conversion of pyruvate to LA mediated by LDHA (44). In addition, VTE can result in glucose and oxygen deficiency in local VECs, leading to changes in the levels of metabolic substances, including LA, which plays an important role in VTE (45, 46). Therefore, LDH may affect VEC supply capability by influencing lactic acid metabolism. This, in turn, could impact the prognosis of patients with VTE. However, the specific mechanism by which LDH is involved in the occurrence and adverse prognosis of VTE remains unclear. Further research is needed to elucidate this mechanism.

ALB is an acute-phase protein primarily synthesized in the liver and serves as a reliable indicator of nutritional status (18). It is the most abundant circulating protein endowed with multiple functions, including transport of molecules, maintenance of colloidal osmotic pressure, anti-inflammatory and antioxidant properties, and inhibitory activity toward the clotting system and platelet activation (19). A low ALB concentration is considered an integrative index for inflammation, hypercoagulability, or disease states that predispose patients to thrombosis (47). The mechanisms by which low ALB is associated with VTE may include the following aspects: first, ALB is considered an acute-phase inflammatory response protein with crucial physiological functions in endothelin stabilization and inflammatory pathways (48). Inflammation may result in endothelial damage in the venous wall, exposure of collagen, and ultimately thrombosis. This inflammatory response in the venous wall can further stimulate inflammation (49). In addition, inflammation can increase vascular permeability, leading to additional loss of ALB. Second, low albumin levels can contribute to a hypercoagulable state in the blood. Reduced ALB has been associated with higher fibrinogen and factor VIII (FVIII) levels, as well as a shorter activated partial thromboplastin time (aPTT) (20). This suggests that low ALB may indicate a tendency toward hypercoagulation. ALB plays a crucial role in maintaining plasma colloid osmotic pressure. As ALB levels decrease, water leaks out of blood vessels, causing increased blood viscosity, slowed flow rate, elevated shear force between blood and vessel endothelium, and increased susceptibility of vessel endothelium to injury, ultimately leading to thrombosis (50). Third, ALB significantly contributes to binding anticoagulant factors, inhibiting platelet accumulation. It exerts anticoagulant effects by inhibiting fibrin polymerization and platelet aggregation. ALB also has heparin-like effects by enhancing the activity of antithrombin III (51–53). Moreover, ALB demonstrates antithrombotic effects by binding nitric oxide (NO) at the Cys-34 site, forming nitroso-albumin. This compound appears to prevent the rapid inactivation of NO, thereby prolonging its vasodilating and anti-aggregation effects on platelets (54, 55). Finally, low albumin levels result in increased bioavailability of arachidonic acid and elevated thromboxane A2, favoring platelet aggregation and hyperactivity (52). Jellinge et al. (21) and Thongprayoon et al. (22) found hypoalbuminemia to be associated with short- and long-term mortality in hospitalized patients. In a population-based prospective cohort study including 109 patients with VTE, ALB was found to potentially influence the risk of future VTE (25). Furthermore, a study analyzing 552 patients with acute PE found that ALB was associated with acute PE severity and that patients with hypoalbuminemia had an increased risk of VTE (20). Olson et al. (23) and Kunutsor et al. (25) found the same result. In addition, Königsbrügge et al. carried out a prospective observational cohort study and found that decreased serum albumin levels in patients with cancer were significantly associated with an increased risk of VTE and mortality (24). Gök et al. conducted a retrospective study and found that low ALB levels (OR = 0.049, 95% CI: 0.006–0.383; *p* = 0.049) were associated with 30-day mortality in patients with acute PE (26). Hoskin et al. found that hypoalbuminemia independently predicted both 30-day (OR = 2.57, 95% CI: 1.03–6.41) and 90-day (HR: 2.42 95% CI: 1.38–4.22) mortality. In our study, we found a significant association between LAR and 30-day mortality in patients with acute PE, even after adjusting for multiple factors.

While this study presents compelling evidence supporting the predictive value of LAR for 30-day all-cause mortality in patients with acute PE using a comprehensive database, it is important to acknowledge its limitations. First, the use of ICD-9 and ICD-10 codes for identifying acute PE or other comorbidities introduces a potential source of partial error. Second, the single-center, retrospective nature of our study may lead to selection bias. Due to a large amount of missing data, this study ultimately only included patients with acute PE patients in the ICU, where a high prevalence of comorbidities was observed. This may have had some impact on the study's conclusions. However, we conducted a sensitivity analysis, including non-ICU-managed individuals, and the results remained robust. Furthermore, given our study's limited sample size and the inability to classify the cause of death, we solely explored in-hospital mortality. This might result in an overestimation of the overall outcome. Future research with a multicenter, prospective design is crucial to validate our findings. Third, the absence of some crucial data, such as echocardiography, D-dimer, troponin, and N-terminal pro-BNP, which are vital indicators for assessing acute PE, poses a limitation. Further randomized controlled trials are necessary for validation in the future. Fourth, despite our efforts to adjust for known confounding factors using multivariate analysis, the presence of residual confounding by unknown factors cannot be ruled out. Finally, we did not explore the impact of LAR on long-term mortality due to the lack of long-term mortality data in the included studies.

## Conclusion

This study evaluated the predictive values of LAR for 30-day mortality of patients with acute PE. We found that LAR was a potential prognostic biomarker for predicting 30-day mortality in patients with acute PE, which might help clinicians enhance risk stratification, design individual treatments, and determine follow-up schedules for these patients. This study provides some suggestions for the risk stratification of patients with acute PE.

## Data Availability

The raw data supporting the conclusions of this article will be made available by the authors, without undue reservation.
